# Psychological working conditions and predictors of occupational stress among nurses, Salaga Government Hospital, Ghana, 2016

**DOI:** 10.11604/pamj.2019.33.320.16147

**Published:** 2019-08-23

**Authors:** Basil Benduri Kaburi, Fred Yaw Bio, Chrysantus Kubio, Donne Kofi Ameme, Ernest Kenu, Samuel Oko Sackey, Edwin Andrew Afari

**Affiliations:** 1 Ghana Field Epidemiology and Laboratory Training Programme, Department of Epidemiology and Disease Control, School of Public Health, University of Ghana, Legon, Ghana; 2 Bramuo Medical Centre, Abrankese, Ashanti, Ghana; 3 Ghana Health Service, District Health Directorate, Soboba, Northern Region, Ghana

**Keywords:** Nurses, occupational stress, predictors, psychological working conditions Salaga Government Hospital, Ghana

## Abstract

**Introduction:**

Occupational stress is a recognized health problem among nurses. Globally, its prevalence varies between 9.2% and 68.0%. It detracts from nurses' quality of life and efficiency of job performance. In Ghana, we do not know the important contributory factors to this problem. Our study sought to identify the important predictors of occupational stress among nurses.

**Methods:**

In January 2016, we conducted an institutional-based survey among nurses of Salaga Government Hospital. They completed a five-point Likert type questionnaire adopted from the British Psychological Working Conditions Survey, and the Nurse Stress Index. Across 30 predictor variables, a mean score of 4.00 to 5.00 represented high to extreme occupational stress. We performed bivariate and multivariate analyses to identify important predictors of occupational stress at 95% confidence level.

**Results:**

Of 167 nurses, 58.1% (97) were females. Respondents who experienced high to extreme stress levels had a 2.3 times odds of reporting sickness absence (CI: 1.03-5.14). Sources of occupational stress included: manual lifting of patients and pieces of equipment (OR: 16.23; CI: 6.28 - 41.92), the risks of acquiring infections (OR: 14.67; CI 5.90 - 36.46), receiving feedback only upon unsatisfactory performance (OR: 28.00; CI: 9.72 - 80.64), and inadequate opportunities for continuous professional development (OR: 63.50; CI: 19.99 - 201.75).

**Conclusion:**

The working conditions of nurses were stressful. The most significant predictors of occupational stress were poor supportive supervision by superiors, lack of adequate skills to perform routine tasks, uncertainty about their job role, and the lack of adequate opportunities for career advancements.

## Introduction

Stress is the psychological and physiological response to undesirable experiences generally termed as stressors [1]. Though “stress” is more commonly thought of as harmful, responses to stress are a spectrum that stretch from the less discussed “eustress” - where positive responses such as innovation and improved productivity result, to “distress” - which is associated in varying degrees to the better known negative outcomes of stress [2]. An individual's stress threshold is influenced by the source of stress, their personal characteristics, experiences, and coping skills [3]. Stress, stress-related diseases, and their ensuing disabilities are prevalent the world over [4].

Four main sources of stress have been described viz. the physical environment, social stressors, physiological, and psychological [5]. In varying degrees of importance, all these sources of stress prevail in various occupational settings [6]. Occupational stress results from a perceived imbalance between workplace stressors and coping abilities of the worker; leading to negative health outcomes [7]. According to the American Institute of Stress, about eight of ten occupational injuries, and four of every ten employee turnovers are largely stress related [8]. Occupational stress has also been implicated in the aetiologies of anxiety and depressive symptoms [7,9].

Occupational stress is a recognized health problem among nurses [10]. Globally, its prevalence among nurses varies widely between 9.2% and 68.0% [5]. An interplay of several factors at the workplace contributes to occupational stress among nurses. Key among these factors are: excessive workloads, the need for a constant attention to details of patient care, dealing with both physically and emotionally exhausting situations, the lack of adequate autonomy for decision making, and low levels of cooperation from patients and their relatives [4,11]. Furthermore, work settings and the sociocultural orientation of nurses have been reported to influence thresholds for developing stress across communities and countries [12].

Occupational stress detracts from nurses' quality of life and efficiency of job performance [10]. Nursing related stress also contributes to absenteeism and high turnover rates in the profession [13]. Stressed nurses tend to be apathetic towards patients, thereby increasing their error rates in administering treatments [14]. The results are poor patient care, poor disease outcomes, and increased cost of healthcare services [15].

Assessing this subjective phenomenon of stress is bound to be difficult, and particularly so in an occupation such as nursing that requires diverse skills, team work, concentration, and emotional control. Yet, in order to tackle this problem among nurses, a good understanding of their sources of stress is non-negotiable. Compared to their counterparts in developed countries, nurses in developing countries such as Ghana are especially disadvantaged in their working conditions viz. low salaries, excessive workloads with unpaid extra hours of work, poor hospital infrastructure (working environment), inadequate resource to perform duties, and fewer opportunities for career development [16]. These factors induce low morale; a precursor to occupational stress.

In Ghana, occupational stress and its sources among nurses is largely under-explored by researchers. Consequently, evidence on the important sources of occupational stress among nurses in Ghana is scarce. We conducted this study to assess the psychological working conditions, and to identify the important predictors of occupational stress among nurses in Salaga Government Hospital.

## Methods

### Study area

The study was conducted in Salaga Government Hospital. It is a primary healthcare facility located in Salaga; one of the rural districts in Northern Ghana. Based on a population of 135,000 from the 2010 census, Salaga Government Hospital serves a projected population of about 170,000 people within its catchment area [17]. Services offered by the hospital include: outpatient consultation, in-patient care, general surgeries, obstetric and gynaecological services, antenatal and postnatal care, biomedical and radiological diagnostic services. It also serves as a referral center for smaller health facilities; in and around the district.

The average nurse in the hospital is expected to work a maximum of seven hours for day duties, and twelve (12) hours for night shifts. Depending on the working unit and the prevailing staff strength, night shifts run for four (4) to seven (7) days followed by three (3) to five (5) days off duty. The working environment is typical of a deprived district hospital in Ghana where infrastructural expansion took place in piecemeal over time; leading to structures that are poorly adapted for well-coordinated work. As a deprived district hospital, it does not attract the full complement of skilled health workers such as doctors and nurses. It is served by one doctor who doubles as the head of the hospital management team. As a result, nurses often step in to perform tasks outside their job description. The head of nurses in the hospital is the nurse manager who is assisted by ward and department heads in the day to day administration of nursing services. The hospital does not have an occupational health unit to see to the occupational health needs of its staff.

### Operational definition of occupational stress

This is the harmful physical and emotional responses that occur when a worker's capabilities, resources, or needs do not match the job demands.

### Study design

The study was an institutional-based survey.

### Population

The source population was all nurses of Salaga Government Hospital. The study population was all nurses of Salaga Government Hospital who were available in the facility during the data collection period.

### Inclusion and exclusion criteria

All nurses in three categories (state registered nurses, midwives, and enrolled nurses) who were working in the hospital at the time of data collection and consented to participate in the study were included. Nurses on pre-appointment orientation were excluded from the study.

### Study variables

*Independent variables:* socio-demographic characteristics age, sex, marital status, type of nursing qualification, length of service, presence of chronic disease; respondents' perceptions of potential sources of stress such as: workload pressures related to patient load, job risks and resource constraints, organizational support and involvement, dealing with patients and relatives, home and work conflicts, and confidence and competence in job.

*Dependent variable:* occupational stress among nurses is the dependent variable. It is an ordinal qualitative variable classified at two levels as: low to moderate occupational stress versus high to extreme occupational stress.

### Data collection instrument

In January 2016, we collected data over a period of four weeks using a self-administered questionnaire adapted from British Psychological Working Condition Survey, and the Nurse Stress Index. The data collected were: socio-demographic characteristics, perceived impact of work on physical and mental health, work related sickness absence, and predictors of occupational stress. The predictors of occupational stress were grouped into six (6) subscales under the Nurse Stress Index, each consisting of five items. The sub-scales included: workload pressures related to patient load, (items 1-5); job risks and resource constraints, (items 6-10); organizational support and involvement, (items 11-15); dealing with patients and relatives, (items 16-20); home and work conflicts, (items 21-25); and confidence and competence in role, (items 26-30). Respondents rated these predictors of occupational stress on a 5-point Likert scale ranging from 1 = no stress to 5 = extreme stress. For each item, the respondents rated their perception by ticking an applicable score as follows: 1 = no stress at all, 2 = very little stress, 3 = moderate stress, 4 = high stress, and 5 = extreme stress. The scores derived from each subscale were compared directly to obtain information on the relative importance of each subscale as a source of occupational stress to the nurses.

### Data quality control

Data were entered into Ms-Excel version 2010 and assessed for completeness, consistency, and missing values. Two questionnaires that were incomplete were excluded from the analysis.

### Data analysis

The cleaned data from excel were imported to STATA 13.1 version for analysis. We performed summary descriptive statistics; including the computation of percentage mean scores for each of the six (6) stress subscales using the following formula: *(Actual Computed Mean Score / Maximum Potential Mean Score) X 100%*.

The percentage mean scores were used to compare the relative importance of the six subscales on occupational stress among the nurses. Absolute mean scores were computed for each respondent by summing scores of the 30 items on potential predictors of occupational stress and dividing by 30. For each respondent a mean score range of 1.00 to 3.00 across the 30 predictor variables represented low to moderate stress, and a mean score range of 4.00 to 5.00 represented high to extreme stress. All statistical analysis were performed at 95% confidence level. We used bivariate logistic regression to determine variables that were independently predictive of high to extreme occupational stress. We then used the stepwise backward elimination process to enter selected variables into a multiple logistic regression model based on positive association with outcome variable with a p < 0.10 at the preliminary bivariate analysis. In the resulting model, statistical significance for predictors of high to extreme occupational stress was set at p < 0.05.

### Ethical considerations

The study was conducted as part of a health needs assessment with institutional permission from the hospital health management team. It was an in-service research aimed at improving the health of staff and quality of care for patients. The nurses understood that the study was optional, and they could withdraw summarily from it at any point without any sanctions. They also understood that, participation in the study did not come with any personal gains; financial or otherwise. To maintain confidentiality, respondents understood and complied with the instructions that personal identifiers were not accepted on the questionnaire sheets, and that completed questionnaires were to be returned in sealed envelopes that were provided. These were deposited at the hospital administration and opened at the end of the data collection period.

## Results

### Socio-demographic and work related characteristics of respondents

A total of 189 eligible nurses were invited to participate. Of these, 167 returned completed questionnaires giving a response rate of 88.4%. The median age of respondents was 32 years (range 19 – 62 years). Majority of the respondents; 58.1% (97) were females. Nearly half of respondents 49.7% (83) were married, and 46.1% (77) had never married (Table 1).

**Table 1:** Socio-demographic characteristics of nurses, Salaga Government Hospital, 2016 (n=167)

Variable	Classification	Number	Proportion (%)
**Age**	≤ 24	32	19.2
	25-34	66	39.5
	35-44	39	23.4
	45-54	19	11.4
	55-64	11	6.6
**Sex**	Male	70	41.9
	Female	97	58.1
**Disability**	Present	3	1.8
	Absent	164	98.2
**Chronic disease**	Present	21	12.6
	Absent	146	87.4
**Marital status**	Married	83	49.7
	Single	77	46.1
	Separated/divorced	4	2.4
	Widowed	3	1.8
**Working unit**	OPD	20	12.0
	Paediatric Ward	42	25.1
	Surgical Ward	39	23.4
	Medical Ward	35	21.0
	Maternity Ward	31	18.6
**Nurse category**	SRN	49	29.3
	Midwife	7	4.2
	Enrolled	111	66.5
**Years** **of practice**	1-5	66	39.5
	6-10	31	18.6
	11-15	23	13.8
	16-20	15	9.0
	> 20	32	19.2

### Psychological working conditions of respondents

**Figure 1:**
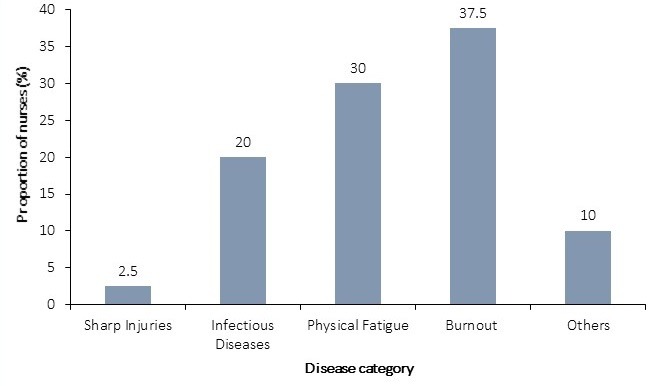
Categories of diseases implicated for sickness absence among nurses, Salaga Government Hospital, 2016

**Figure 2:**
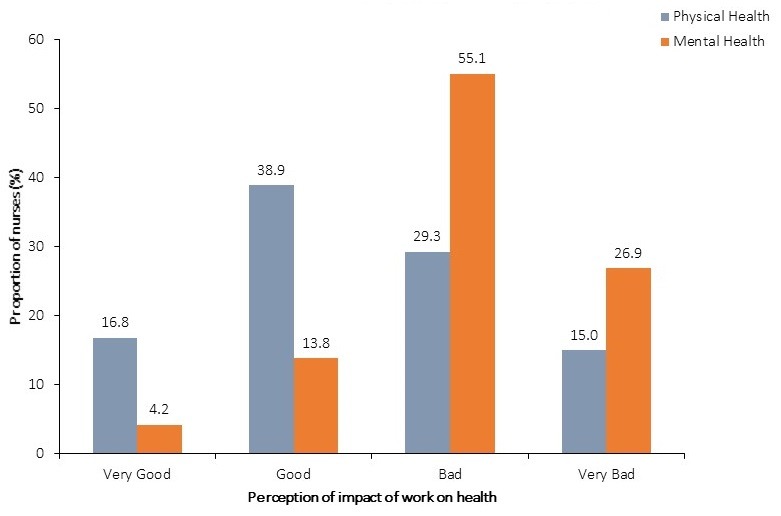
Nurses’ perceived impact of work on their health, Salaga Government Hospital, 2016

Of the 167 nurses, 35 (21.0%) experienced high to extreme levels of occupational stress. All the nurses worked an average of 45 ± 3.7 hours each week. In the year preceding the assessment, a total of 82 working days were lost to sickness absence from 40 nurses. Compared to nurses who had mild to moderate stress levels, nurses who experienced high to extreme stress levels had a 2.3 times odds of reporting sickness absence (CI: 1.03-5.14). Burnout was the leading condition (37.5%) implicated in sickness absences among the nurses (Figure 1). Whereas 44.3% (74) of respondents perceived that their work adversely impacted their physical health, 82.0% (137) perceived the mental health was more adversely impacted by their work (Figure 2).

### Predictors of occupational stress among respondents

Of the six (6) subscales, workload produced the highest percentage mean score of 79.66% (Table 2).

**Table 2:** Percentage mean scores of occupational stress subscales among nurses, Salaga Government Hospital, 2016

Stress Subscale	Mean score	Std. Dev.	%Mean Score (%)	Minimum (%)	Maximum (%)
Workload (patient load)	3.98	1.11	79.66	20	100
Job risks and resource availability	2.38	1.13	47.54	20	100
Organizational support and involvement	2.30	1.15	46.04	20	100
Dealing with patients and relatives	3.73	1.01	74.51	20	100
Home and work conflicts	2.26	1.16	45.17	20	100
Confidence and competence in role	2.38	1.20	47.57	20	100

From a bivariate analysis, all the predictors had positive associations (OR crude > 1) with high to extreme levels of occupational stress among the nurses (Table 3, Table 4, Table 5 ). However, six (6) of these associations viz. inadequate time for work volume, time demands from peers, emergencies, shift work, aggressive people, and dealing with the dying patient, were not statistically significant at 95% confidence level.

**Table 3:** Bivariate logistic regression for workload and job related risk predictors of occupational stress levels among nurses, Salaga Hospital, 2016

Predictor Variables	Mild to moderate stress n(%)	High to extreme stress n(%)	OR _crude_(95% CI)	p-value
**Time is adequate time for work volume**				
Yes	16(9.58)	116(69.46)	1	
No	1(0.60)	34(20.36)	4.69(0.60-36.65)	0.141^#^
**Peers do not demand much of my time**				
Yes	15(8.98)	117(70.06)	1	
No	2(1.20)	33(19.76)	2.12(0.46-9.72)	0.336^#^
**Trivial tasks do not stress me much**				
Yes	99(59.28)	33(19.76)	1	
No	9(5.39)	26(15.57)	8.67(3.69-20.36)	< 0.001
**Emergencies do not stress me much**				
Yes	32(19.16)	100(59.88)	1	
No	3(1.80)	32(19.16)	3.41(0.98-11.90)	0.054^#^
**Shift work does not stress me much**				
Yes	12(7.19)	120(71.86)	1	
No	1(0.60)	34(20.36)	3.40(0.43-27.09)	0.248^#^
**Risks of infections do not stress me much**				
Yes	121(72.45)	11(6.59)	1	
No	15(8.98)	20(11.98)	14.67(5.90-36.46)	< 0.001
**Resource shortages do not stress me much**				
Yes	123(73.65)	9(5.40)	1	
No	21(12.57)	14(8.38)	9.11(3.50-23.72	< 0.001
**Manual lifting does not stress me much**				
Yes	123(73.65)	9(5.39)	1	
No	16(9.58)	19(11.38)	16.23(6.28-41.92)	< 0.001
**The physical working environment is safe**				
Yes	121(72.46)	11(6.58)	1	
No	19(11.38)	16(9.58)	9.26(3.74-22.95)	< 0.001
**Management does not interrupt work**				
Yes	123(73.65)	9(5.39)	1	
No	22(13.17)	13(7.78)	8.08(3.08-21.16)	< 0.001

**Table 4:** Bivariate logistic regression for organizational, patient and relative predictors of occupational stress levels among nurses, Salaga Hospital, 2016

Predictor Variables	Mild to moderate stress n(%)	High to extreme stress n(%)	OR _crude_(95% CI)	p-value
**I am often involved in decision making**				
Yes	79(47.30)	53(31.74)	1	
No	11(6.59)	24(14.37)	3.25(1.47-7.19)	0.004
**Management understands the needs of my department**				
Yes	128(76.65)	4(2.39)	1	
No	23(13.77)	12(7.19)	16.70(4.95-56.30)	< 0.001
**Peers are supportive with work challenges**				
Yes	123(73.65)	9(5.39)	1	
No	19(11.38)	16(9.58)	11.51(4.46-29.72)	< 0.001
**Feedback only on unsatisfactory performance does not stress me much**				
Yes	126(75.45)	6(3.59)	1	
No	15(8.98)	20(11.98)	28.00(9.72-80.64)	< 0.001
**I receive supportive supervision from superiors**				
Yes	130(77.84)	2(1.20)	1	
No	17(10.18)	18(10.78)	68.82(14.67-322.91)	< 0.001
**Aggressive people do not stress me much**				
Yes	47(28.14)	85(50.90)	1	
No	8(4.79)	27(16.17)	1.87(0.79-4.44)	0.158^#^
**Difficult patients do not stress me much**				
Yes	129(77.24)	3(1.80)	1	
No	16(9.58)	19(11.38)	51.06(13.59-191.88)	< 0.001
**Life and death situations do not stress me much**				
Yes	40(23.95)	92(55.10)	1	
No	5(2.99)	30(17.96)	2.61(0.94-7.21)	0.065^#^
**Counselling bereaved patient relatives does not stress me much**				
Yes	64(38.32)	68(40.72)	1	
No	9(5.39)	26(15.57)	2.72(1.18-6.24)	0.018
**Physical and verbal abuses from patient relatives do not stress me much**				
Yes	31(18.56)	101(60.48)	1	
No	2(1.20)	33(19.76)	5.06(1.15-22.31)	0.032

**Table 5:** Bivariate logistic regression for home, work, and role conflicts related predictors of occupational stress levels among nurses, Salaga Hospital, 2016

Predictor Variables	Mild to Moderate Stress n(%)	High to Extreme Stress n(%)	OR _crude_ (95% CI)	p-value	
**Social relations outside work do not cause me much stress at work**					
**Yes**	123 (76.65)	9 (5.39)	1		
**No**	13 (7.78)	22 (13.17)	23.13 (8.83 – 60.61)	< 0.001	
**Superiors quite appreciate my home pressures**					
**Yes**	124 (74.25)	8 (4.79)	1		
**No**	14 (8.38)	21 (12.58)	23.23 (8.69 -62.20)	< 0.001	
**I do not have much difficult balancing home and job demands**					
**Yes**	121 (72.45)	11 (6.59)	1		
**No**	20 (11.98)	15 (8.98)	8.25 (3.32 – 20.51)	< 0.001	
**Family supports me enough with job demands**					
**Yes**	130 (77.84)	2 (1.20)	1		
**No**	17 (10.18)	18 (10.78)	68.82 (14.67 – 322.92)	< 0.001	
**Absenteeism due to home demands does not stress me much**					
**Yes**	128 (76.65)	4 (2.40)	1		
**No**	14 (8.38)	21 (12.57)	48.00 (14.41 – 159.88)	< 0.001	
**I am able to contribute to some change in the hospital**					
**Yes**	119 (71.26)	13 (7.78)	1		
**No**	11 (6.59)	24 (14.37)	19.97 (8.00 – 49.86)	< 0.001	
**I am able to use much of my knowledge and skills**					
**Yes**	106 (63.47)	26 (15.57)	1		
**No**	19 (11.38)	16 (9.58)	3.43 (1.56 – 7.58)	0.002	
**Inadequate CPD training opportunities does not stress me much**					
**Yes**	127 (76.05)	5 (2.99)	1		
**No**	10 (5.99)	25 (14.97)	63.50 (19.99 – 201.75)	< 0.001	
**Lack of specialized training for present tasks does no stress me much**					
**Yes**	131 (78.44)	1 (0.60)	1		
**No**	15 (8.98)	20 (11.98)	174.67 (21.86 -1395.61)	< 0.001	
**Uncertainty about my job description does not stress me much**					
**Yes**	130 (77.84)	2 (1.20)	1		
**No**	6 (3.59)	29 (17.37)	314.17 (60.33 – 1636.09)		<0.001

^#^ Association is not statistically significant

On the workload stress subscale, duties that are not directly related to patient care (trivial duties) were a significant source of stress (OR: 8.67; CI: 3.69 - 20.36). On the subscale of job risks and resource availability, the leading predictors of stress were: manual lifting of patients and pieces of equipment (OR: 16.23; CI: 6.28 - 41.92) and the risks of acquiring infections (OR: 14.67; CI 5.90 - 36.46). On the subscale of organizational support for respondents, inadequate support from superiors (OR: 68.82; CI: 14.67 - 322.91), and receiving feedback only upon unsatisfactory performance (OR: 28.00; CI: 9.72 - 80.64), were important sources of stress. On the remaining subscales, significant predictors of stress included: inability to contribute to change in the hospital (OR: 19.97; CI:8.00 - 49.86), Inadequate opportunities for continuous professional development (CPD) (OR: 63.50 CI: 19.99 - 201.75), lack of specialised skills for current tasks (OR: 174.67; CI: 21.86 -1395.61), and uncertainty about their job description (OR: 314.17; CI: 60.33 - 1636.09).

On multiple logistic regression (Table 6), significant predictors of occupational stress were: inadequate training opportunities (OR adjusted: 68.18; CI: 2.28 - 2035.4), and receiving feedback from superiors only on occasions of unsatisfactory performance (OR adjusted: 6.49; 2.80 - 5079.90). With a total of eight (8) predictors, the model had a probability of 86.57% of predicting high to extreme occupational stress; and an overall significant fit (p < 0.0001). The model predicted high to extreme occupational stress levels 86.57% of the time.

**Table 6:** Multiple logistic regression model for predicting high to extreme occupational stress levels among nurses, Salaga Hospital, 2016

Predictor Variables	OR _adjusted_	95% CI	p-value
Uncertainty about job description	18.59	0.97-357.35	0.053
Lack of specialized skills for current tasks	4.61	0.02-1339.90	0.597
Inadequate support from superiors	44.55	0.35-5615.40	0.124
Lack of family support	16.46	0.03-9258.00	0.386
Inadequate training opportunities	68.18	2.28-2035.40	0.015 *
Dealing with difficult (uncooperative) patients	6.81	0.22-207.06	0.271
Absenteeism due to home pressures	6.49	0.21-197.45	0.283
Feedback for only unsatisfactory performance	6.49	2.80-5079.90	0.013*

n=167, LR χ^2^ = 148.44, p < 0.0001, Pseudo R^2^ = 0.8657.

* significant predictors of high to extreme stress*

## Discussion

Our study assessed the conditions under which nurses of Salaga Government Hospital worked. We also attempted to identify a set of stressors that predict extreme levels of stress among them. Results from the bivariate logistic regression revealed that 24 of the 30 potential predictors of occupational stress had statistically significant positive associations with high to extreme levels of occupational stress (p <0.05) On a multiple logistic regression analysis, lack of opportunities to advance career, and receiving feedback only on occasions of unsatisfactory performance were each significantly predictive of high to extreme levels of occupational stress among our respondents.

The study results revealed that a high proportion of the nurses perceive that their psychological wellbeing is adversely affected by various aspects of their job. About two out of every ten of them perceive they are either highly or extremely stressed by their work. About twice the proportion of nurses (82.0%) perceive that work related stress impact more adversely on their mental health compared to their physical health (44.3%). Irrespective of its mode of manifestation, occupational stress is a recognized cause of sickness absence among employees. In a systematic review of the literature on work related ill health and sickness absence, Michie and Williams found that, psychological ill health impacted so severely on nurses' health that it accounted for most short term sick leaves [18]. In our study, the leading cause of sickness absence was job related burnout; a severe form of stress that manifests as physical and emotional exhaustion following a poorly managed stress. Among our respondents, our findings revealed that nurses who experienced high to extreme levels of occupational stress were over two times more likely to miss work on account of sickness absence (OR: 2.3; CI: 1.03 - 5.14). This association between job stress and sickness absence among nurses was also found among some a nurses in the province of Quebec where sickness absence was a leading consequence of job strain [19].

Based on descriptive analysis of our study data using percentage mean scores, two of the six subscales viz. workload/patient load (%mean score = 79.66) and dealing with patient relatives (%mean score = 74.51) were the leading general sources of occupational stress. Of the ten specific potential predictors of stress under these two subscales, five of them were particularly important - shift work, inadequate time for work volume, abuses from patient relatives, time demands by colleagues, and having to deal with life and death situations. A low nurse-patient ratio contributes to poorly rationalized shifts. In particular, long term night shift work puts the nurses at an increased risk of cardiovascular diseases [20,21]. Excessive workloads can render these nurses apathetic towards patients and their relatives [15]. This situation, coupled with longer waiting times, frustrates patient relatives who sometimes express their frustrations by verbally and/or physically abusing nurses. With overwhelming workloads and frequent request for assistance from other over-worked nurses, patient care is hardly optimum, and their disease outcomes are bound to be sub-optimal also [3,14]. In a descriptive correlational cross sectional study of nurses' perceived job related stress and job satisfaction in Amman private hospitals in Jordan, an insufficient number of nurses resulting in excessive workloads was among the leading causes of perceived occupational stress [22].

In our study, compared to those who do not undertake trivial tasks (dusting, mobbing floors, picking medicines from the pharmacy), those who did were over 8 times more likely to experience high to extreme levels of stress. It has been suggested that, to reduce workload of nurses, staffing levels of both nurses and administrative staff must be increased, and more of the paper work delegated to the administrative staff [23]. By extension, though these trivial tasks are traditionally part of nursing duties, delegating such tasks to another cadre of staff should take some workload off the nurses. In China also, a study by Wu and colleagues on the relationship between burnout and occupational stress among nurses, work overload was reported as an important source of stress [24].

Dealing with terminally ill patients and dying patients is a source of stress among our respondents. In particular, counselling bereaved patient relatives has about a threefold significant risk (CI: 1.18 - 6.24) of stressing up our respondents. This finding is in keeping with findings from a cross sectional study on job related stress among nurses working in some public hospitals in Ethiopia, where an overall job related stress resulted from dealing with death and dying patients [5]. Also, in an assessment by Makie of stress and coping strategies amongst registered nurses in a tertiary hospital in South Africa, emotional issues surrounding death and the dying patient was perceived by respondents to be one of the most stressful aspects of their work [25].

Among our respondents, those who experience verbal and physical abuse from patient relatives have a five-fold significant risk (CI: 1.15 - 22.31) of experiencing high to extreme stress levels. Such abuses have been reported as an important source of stress for nurses in Turkey [26]. Bakker and colleagues assert that instead of being stressful, helping the sick should be personally gratifying, except when patients and their relatives do not appreciate efforts nurses make to care for them [27]. In a study that explored the sources of verbal abuses suffered by nurses, verbal abuse from patient relatives was found to be second (25%) only to verbal abuse of nurses by other nurses (27%) [28].

Inadequate opportunities for our respondents to advance their career and skills through continuous professional developments had a significant influence in their perception of high to extreme levels of stress. After controlling for confounders, the unfulfilled desire of respondents to advance their careers remained a significant predictor of high to extreme levels of stress. In their study of leadership, organizational stress, and emotional exhaustion among hospital nursing staff in Belgium, Sabine and colleagues found a negative correlation between intellectual stimulation and stress [29]. Inability to update their skills in the face of fast changing work demands is an agreeable reason for occupational stress perception. Our findings also show that, lack of specialized training for present tasks and uncertainty about job description (also termed role ambiguity) were strongly associated with high to extreme levels of occupational stress. These findings are not unexpected because the hospital has only one doctor and most of these nurses step in to perform duties such as minor surgeries and procedures which should have been performed by a doctor.

Nearly half (16/35) of our respondents who experienced high to extreme levels of stress perceived that their physical working environment was not safe; and had a nine fold risk (CI: 3.74 - 22.95) of experiencing these levels of stress. In our study, manual lifting of patients and pieces of equipment, and the risk of exposure to infections are particularly associated with increased odds of 16.23 (CI: 6.28 - 41.92), and 14.67 (CI: 5.90 - 36.46) respectively of making nurses perceive high to extreme stress levels (Table 3). In a study on nurse work environment and occupational safety among a cross section of nurses in the United States of America, findings strongly suggested that monitoring nurses' working conditions and improving participatory management of hospitals improves nurse safety, increases financial returns through low absenteeism and turnover rates, and ultimately improves the quality of patient care [30].

Among our respondents, organizational support, poor working relations and inadequate support from superiors (OR: 68.82; CI: 14.67 - 322.91), and receiving feedback only upon unsatisfactory performance (OR: 28.00; CI: 9.72 - 80.64), were important sources of stress. On the remaining subscales, significant predictors of stress included: inability to contribute to change in the hospital (OR: 19.97; CI: 8.00 - 49.86), our respondents' stress levels are positively associated with poor working relations and inadequate support from their immediate superiors. They do not get feedback from their supervisors except on occasions of poor performance. This state of affairs makes respondents feel that their superiors do not acknowledge creditable performances; instead, they only look out for faults so they can reprimand subordinates. Organizational research on the determinants of employees' job-related outcomes illustrates that supervisors may have a significant influence on subordinates' personal and professional outcomes [31]. In the field of nursing, related research findings have shown that immediate supervisors of nurses can mitigate the effects of a demanding work environment on their subordinates by consciously adopting a supportive supervision tailored to the individual needs of their nurses. This could be a main way by which head nurses can reduce work stress among their subordinates. Adequate feedback including acknowledgement of nurses' efforts towards patient care, support from colleagues and superiors, as well as increased career advancement opportunities have been reported to serve as good buffers against occupational stress among nurses [27,32,33]. According to the influential Job Demands-Control (JD-C) model, job related stress is expected to result from high job demands and low job control as well as an interaction between both job characteristics. An inclusive organisational leadership that allows nurses to participate in work scheduling and the determination of acceptable methods of performing some tasks makes them less vulnerable to occupational stress [34].

### Study limitations

First, the data obtained and analysed in this study are based on evaluations of subjective responses. Though most of the nurses participated, sample size was still small. As such some of the confidence intervals were very wide and hence the estimation of the strength associations of predictors to the outcome (high to extreme stress) could be exaggerated. Second, the generalizability of the findings of this study is limited to primary level hospitals like Salaga Government Hospital that are situated in rural areas. Therefore, the findings may not be generalized to include nurses who work in both public and private hospitals located in urban areas where workplace conditions and staffing strengths are mostly better.

## Conclusion

The working conditions of nurses were stressful and their mental health was worse affected compared with their physical health. Most of the predictors of occupational stress were significantly associated with the high to extreme stress levels. The most significant predictors were poor supportive supervision by nurse managers, lack of adequate skills to perform routine tasks, uncertainty about their job role, and the lack of adequate opportunities for the nurses to advance their careers and skills.

We recommend to the district health management team to identify leadership and management training courses for head nurses in the hospital. The hospital management team in collaboration with the clinical services division of the regional health directorate should consider instituting regular in-service training programmes that are tailored to the additional duties these nurses undertake owing to the lack of adequate numbers of medical officers. The Ghana Health Service should reinforce the policy of granting earlier study leaves with pay to its personnel working in rural setting to ensure that their career advancement is not affected by serving in resource-limited settings. The Ministry of Health should consider setting up occupational health units in all hospitals with trained staff to see to the occupational health needs of staff. We also recommend to the Ghana Health Service to conduct qualitative studies in this subject area in order to obtain more insights on the important determinants of occupational stress among nurses in Ghana team, including adherence counsellors.

### What is known about this topic

- Even with the best of working conditions, nursing is stressful;
- Stress adversely affects the health and performance of nurses and has also been associated with poor patient outcomes;
- Factors known to contribute to occupational stress among nurses include: the physical conditions of the workplace, job demands, role conflicts, working relationships, and managerial practices.

### What this study adds

- One out of every five nurses experience high to extreme levels of stress in their lines of duty at Salaga Government Hospital, a primary health facility in a rural setting in Ghana;
- The most significant predictors of stress were: poor supportive supervision by nurse managers, lack of adequate skills to perform routine tasks, uncertainty about job role, and inadequate career advancement opportunities.

## Competing interests

The authors declare no competing interests.
